# Engineering osteoclast resorption units *via* sacrificial microgels in a bone-on-chip platform

**DOI:** 10.1039/d5lc00682a

**Published:** 2025-11-11

**Authors:** Francisco Conceição, Nuno Araújo-Gomes, Johanna F. A. Husch, Malin Becker, Jeroen J. J. P. van den Beucken, Jeroen Leijten, Liliana Moreira Teixeira

**Affiliations:** a Department of Bioengineering Technologies, Faculty of Science and Technology, TechMedCentre, University of Twente 7522 NB Enschede The Netherlands l.s.moreirateixeira@utwente.nl; b Regenerative Biomaterials, Department of Dentistry, Radboudumc 6525 EX Nijmegen The Netherlands; c Organ-on-Chip Centre Twente, TechMed Centre, MESA+, University of Twente 7522 NB Enschede The Netherlands

## Abstract

Bone remodeling is a tightly regulated process essential for skeletal health, occurring within specialized structures known as bone multicellular units (BMUs). Despite extensive research, current animal models and conventional *in vitro* systems fail to reproduce the spatial and cellular complexity of human BMUs. Moreover, the mineralized nature of bone tissue poses imaging challenges due to its inherent opacity, limiting the ability to monitor osteoclast activity in complex 3D models. Control over the structure of *in vitro* BMU micro-confined niches would enable more effective 3D analysis readouts of osteoclast activity. Herein we developed a BMU-inspired bone-on-chip platform that enables localized, non-invasive analysis of human osteoclast function. Using microfluidic droplet generation, we encapsulated mature osteoclasts in monodisperse, enzymatically degradable dextran–tyramine (Dex–TA) microgels with defined size. These osteoclast-laden microgels were embedded within mineralized collagen hydrogels and selectively degraded to form confined, cell-populated microstructures directly on-chip. In addition, the contrast between the microcavities and surrounding mineralized matrix allowed non-destructive monitoring of matrix degradation using reflection confocal microscopy. Immunocytochemical analysis confirmed the morphological and functional differentiation of osteoclasts within the platform. Furthermore, our results demonstrated increased matrix resorption in response to RANKL treatment, validating the system's capacity to assess osteoclast activity under relevant stimuli. This bone-on-chip model overcomes key limitations of traditional systems by enabling spatial confinement, controlled degradation, and functional readouts of osteoclast behavior in a human-relevant 3D microenvironment. It is thus a versatile tool for studying bone remodeling and has strong potential for application in disease modeling and drug screening for bone-related disorders.

## Abstract 2

A bone-on-chip model integrating encapsulated osteoclasts within biomimetic mineralized collagen was established to recreate simplified bone resorption units. Osteoclast activity was monitored through non-invasive analysis of resorption compartments.

## Graphical abstract

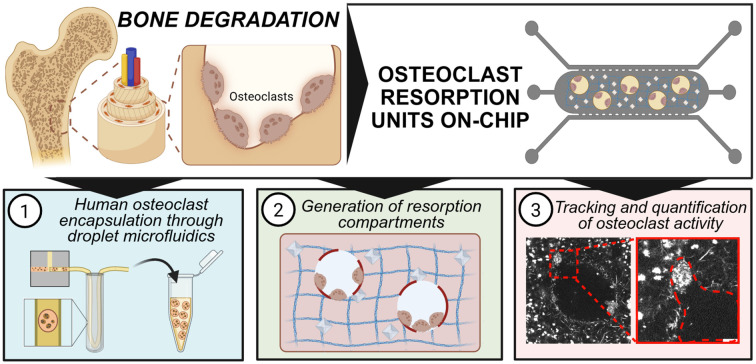

## 1. Introduction

Skeletal structural and functional integrity is maintained through a carefully orchestrated process of bone remodeling, where old or damaged bone matrix is degraded by multinucleated osteoclasts and subsequently replaced by osteoblast-derived mineralized extracellular matrix (ECM).^1^ Bone remodeling is coordinated in specialized structures called bone multicellular units (BMUs), where tightly regulated interactions between osteoclasts and osteoblasts, amongst other cellular components, take place.^1^ In cortical bone, bone remodeling is conducted synchronously in organized BMUs distributed along Haversian canals in close association with neurovascular bundles.^2^ Imbalanced bone remodeling is common in pathological conditions such as osteoporosis, where osteoclast activity is exacerbated leading to increased bone degradation and fracture risk.^3^

Pre-clinical drug development for osteoporosis remains heavily dependent on animal models, but species-specific differences in bone structure and cell metabolism severely limit the translational relevance of these models. As an example, mice do not exhibit cortical bone remodeling in Haversian canals,^4^ thus investigating this process and associated pathologies *in vivo* is challenging. On the other hand, conventional 2D cultures are insufficient to mimic the spatial constraints of bone remodeling, while 3D scaffolds often lack the microstructural precision to generate structures such as the BMU.^5^

Organ-on-chip (OoC) technology has been demonstrating high potential to replace animal testing in drug screening, organ development and pathophysiology, due to the recapitulation of human multicellular architecture, microenvironment and tissue-tissue interfaces leading to higher model accuracy.^6^ One of the main advantages of OoCs is their versatility and the ability to couple with advanced biomimetic materials systems, which offer new avenues to engineer physiologically relevant bone models *in vitro.*^6^ In particular, microgels have emerged as promising tools to recreate structural elements of the extracellular matrix with high control over geometry, mechanical properties and degradability.^7^ Droplet-based microfluidics allows for the production of monodisperse, cell-laden microgels at high throughput, with precise control over droplet dimension and cell density.^8^ The pre-defined size and degradability of microgels render them particularly attractive as sacrificial templates to generate structural features on-demand, directly on-chip.^9^

To date, bone-on-chip platforms have been developed for modeling osteoblastic lineage differentiation and bone regeneration,^10^ the bone marrow compartment,^11^ osteocyte-osteoblast communication,^12^ osteochondral interface,^13,14^ and cancer metastasis,^15^ among others.^10,16,17^ However, despite the crucial importance of osteoclast resorption activity in the onset and development of osteoporosis, osteoclasts are often overlooked in currently available bone-on-chip models. Models that incorporate human osteoclasts are focused on cell differentiation and their interactions with other bone-resident cells, while providing limited insight into their local matrix degradation activity.^18,19^ Generating bioinspired, micro-structured BMU directly on-chip allowing the direct measurement of osteoclast activity would be of high value for both disease modeling and drug development purposes.

Herein, we present a BMU inspired bone-on-chip system with confined microstructures, generated *via* droplet-generators and embedded in mineralized collagen hydrogels, ultimately aimed at assessing human osteoclast degradation activity. We used hollow tyramine functionalized dextran microgels as templates for localized microcavity formation on-demand with concurrent human osteoclast seeding, providing both a structural and functional approximation of the human BMU. We named such constructs as osteoclast resorption units (ORU). The controlled and predefined size of hollow microgels facilitated the tracking and quantification of osteoclast matrix degradation dynamics. Our platform represents a novel integration of biomaterial microengineering and OoC technology to bridge the gap between oversimplified 2D assays and complex *in vivo* models. Overall, this work opens new avenues for the study of osteoclast-driven matrix degradation in both disease modeling and drug screening approaches.

## 2. Materials and methods

### 2.1. Microfluidic chip fabrication

For device fabrication, master molds were micropatterned with SU-8 with a design adapted from other devices previously established in our department.^20^ The device was composed of one cellular compartment measuring 7.5 mm long and 2.4 mm width, separated from perfusion channels 250 μm wide through a row of trapezoid pillars of 80 × 200 μm. Briefly, h100i orientation silicon wafers (Okmetic) were spin-coated with an SU-8100 negative photoresist (Microchem) at 500 rpm for 30 s and ramped up to 1500 rpm for 30 s, so that the average thickness of the SU-8 was of ∼250 μm. The SU-8 photoresist was then patterned by exposure to UV light with a 365 nm longpass filter using an EVG 6200NT mask aligner (EVGroup, Austria). The patterned wafers were then developed in RER600 (Fujifilm) followed by spraying, spinning, and drying. Finally, patterned wafers were washed with isopropanol (IPA) and dried using a stream of nitrogen gas.

Microfluidic chip devices were produced by soft lithography using polydimethylsiloxane (PDMS). Curing agent (Sylgard 184, Dow Corning) and PDMS prepolymer were mixed in a 1 : 10 ratio, degassed, and poured onto SU-8 molds. PDMS was then cured at 65 °C in an oven for 2 h. The following day, the patterned PDMS was peeled from the SU-8 wafer and cut to the defined shape. The cell chamber inlets/outlets and the perfusion (media) inlets/outlets were punched with 1.5 and 1 mm biopsy punchers, respectively. Each PDMS chip was then oxygen plasma-bonded (Cute plasma oven, Femto Science, South Korea) to a glass coverslip and stored at room temperature (RT) until further use. The day before use, chips were incubated at 65 °C overnight to revert to hydrophobic behavior. Prior to cell seeding/hydrogel injection, chips were sterilized under UV light for 15 min.

### 2.2. Osteoclast precursor isolation and differentiation

Human CD14^+^ monocytes were isolated from whole blood of healthy donors. Blood was collected from healthy donors *via* a voluntary donor service, where repeated testing of specific individual donors was not possible. The study did not fall in the scope of the Dutch Medical Research Involving Human Subjects Act. The study was performed in accordance with the guidelines of the Declaration of Helsinki. In agreement with these guidelines, informed consent was obtained from all volunteers. Furthermore, the blood collection procedure was approved by the Medical Ethical Committee of the Hospital Medisch Spectrum Twente. Whole blood was diluted in phosphate buffered saline (PBS, Gibco) and blood components were separated using gradient centrifugation in Ficoll-Paque Plus (Cytiva) using SepMate™ 50 mL tubes (STEMCELL Technologies). Cells were centrifuged at 1200*g* for 10 min at room temperature (RT) and the peripheral blood mononuclear cell (PBMC) interphase was transferred to new 50 mL tubes. PBMCs were washed twice with cold PBS, resuspended in 0.5% biotin-free BSA (Sigma-Aldrich) and 2 mM EDTA in PBS and filtered through a 40 μm cell strainer (Greiner bio-one). PBMCs were then incubated in BD IMag™ anti-human CD14 magnetic particles (BD-Biosciences) and magnetically separated according to the manufacturer's instructions. CD14^+^ cells were seeded in T75 flasks in α-MEM (Gibco) supplemented with 10% FBS (Sigma-Aldrich), 1% penicillin/streptomycin (Gibco) (Complete Medium) and 25 ng mL^−1^ of recombinant human macrophage colony stimulating factor (rhM-CSF, R&D Systems) at 5% CO_2_ at 37 °C in a humidified incubator. After 2 days, the media were changed to α-MEM supplemented with 10% FBS, 1% penicillin/streptomycin, 25 ng mL^−1^ rhM-CSF and 25 ng mL^−1^ recombinant human receptor activator of nuclear factor κB ligand (rhRANKL, R&D Systems) and cells were cultured for up to seven days, changing media twice.

### 2.3. Hollow microgel generation and characterization

A polymethylmethacrylate (PMMA) device was used to generate droplets as previously described.^21,22^ Fused silica capillaries were used as nozzles with 200 μm inner diameter which were inserted into semipermeable LIVEO silicone laboratory tubing (DuPont). A round glass capillary (CM Scientific) was then pushed over the nozzle and stabilized with glue.

Tyramine-conjugated dextran (Dex–TA) was synthesized as reported elsewhere,^23^ and used with a degree of substitution of 13%. Droplets comprised of 5% (w/v) Dex–TA, 40 U mL^−1^ type VI horseradish peroxidase (HRP, Sigma-Aldrich) in PBS were manufactured in an oil phase containing *n*-hexadecane (Sigma-Aldrich) with 1% Span 80 (Sigma-Aldrich). Dex–TA hollow microgels were crosslinked by flowing polymer droplets through 30 cm silicone tubing immersed in 30% H_2_O_2_ at a 40/8 μL min^−1^ (oil/polymer) flow rate. The microgels were collected, and the emulsion was broken by three steps of washing with *n*-hexadecane. For characterization of microgel size and thickness, cell-free microgels were stained with ethidium homodimer 1 (EtHD1) and imaged with a LSM880 confocal microscope (Carl Zeiss AG) at 20× 0.8NA (0.42 μm per pixel). Microgels shell thickness and diameter were measured using ImageJ software (v. 1.54f, NIH).

### 2.4. Osteoclast encapsulation

After 9 days of culture, mature osteoclasts were detached with accutase for 10 min at 37 °C, scraped and collected with Complete Medium. Osteoclasts were then centrifuged and resuspended in 300 μL of hydrogel precursor consisting of 5% (w/v) Dex–TA, 40 U mL^−1^ HRP in serum-free α-MEM at a density of 0.5 or 1 × 10^7^ cells per mL. In addition, 8% OptiPrep™ density gradient medium (Sigma-Aldrich) was added to have a hydrogel precursor solution density of 1.05 g L^−1^.^22^ The cell-laden hydrogel precursor solution was loaded into a gastight syringe and continuously stirred using magnets to prevent cell aggregation. Cell-laden microgels were generated with the same oil phase and flow conditions as the ones used for empty microgels. Emulsion was broken by three steps of washing with *n*-hexadecane and then resuspended in serum-free α-MEM.

### 2.5. Microgel loading in the OoC platform and subsequent enzymatic degradation

Cell-laden microgels were then centrifuged at 100*g* for 3 min and resuspended in 4 mg mL^−1^ collagen I solution (Advanced Biomatrix) supplemented with 0.5% hydroxyapatite (HA, formulated as described elsewhere^24^) and 10 μg mL^−1^ vitronectin (Advanced Biomatrix). The microgel suspension was thoroughly mixed and then 10 μL of suspension were injected into the cellular compartment of each microfluidic chip. Collagen was gelated at 37 °C for 1 h 30 min. Serum-free α-MEM was then injected into the perfusion channels followed by incubation of undiluted TrueGel3D™ enzymatic recovery solution (dextranase, Sigma Aldrich) for up to 1 h until the microgels were degraded. The dextranase solution was then washed with α-MEM supplemented with 25 ng mL^−1^ rhM-CSF and rhRANKL and cultured for one week in static conditions in a humified incubator at 37 °C and 5% CO_2_, changing medium daily.

### 2.6. Diffusion studies

Cell-free Dex–TA hollow microgels were suspended in 4 mg mL^−1^ collagen I solution supplemented with 0.5% HA and 10 μg mL^−1^ vitronectin. The microgel suspension was thoroughly mixed and then 10 μL of suspension was injected into the cellular compartment of each microfluidic chip. Collagen was gelated at 37 °C for 1 h 30 min. PBS was added to each perfusion channel before diffusion tests. Permeability of the hydrogels was tested by injecting 1 mg mL^−1^ fluorescein isothiocyanate–dextran – MW 10 kDa (Sigma-Aldrich) in both perfusion channels. The injection occurred sequentially by pipetting manually the fluorophores into the perfusion channels after hydrogel gelation in the central chamber. Imaging and diffusion quantification was performed using an EVOS FL microscope (Thermo Fisher Scientific) every 15 min for a period of 60 min.

### 2.7. Cathepsin K activity and immunocytochemistry

Specific cathepsin K (CTSK) activity was detected using Magic Red™ Cathepsin K kit (Immunochemistry Technologies LLC) according to the manufacturer's instructions. Briefly, after 4 days of culture, MagicRed CTSK probe working solution was injected in the perfusion channels and incubated for 1 h at 37 °C and 5% CO_2_ protected from light. Microfluidic chips were then washed twice with PBS and imaged. A Zeiss LSM880 confocal microscope was used to acquire *z*-stack images at 20× 0.8NA (0.42 μm per pixel) and 2 μm step size.

For immunocytochemistry, after one week of culture, microfluidic chips were washed twice with PBS and fixated with 4% paraformaldehyde at RT for 1 h, followed by another two washes with PBS. Cells were permeabilized with 0.1% Triton X-100 (Sigma-Aldrich) at RT for 15 min and then blocked with 0.1% BSA for 1 h 30 min. Primary anti-CTSK (1 : 200 dilution, Santa Cruz Biotechnology, sc-48 353) was diluted in 0.1% BSA in PBS and incubated at 4 °C overnight. After incubation, microfluidic chips were washed thrice with 0.1% BSA in PBS and incubated with a secondary antibody Alexa-Fluor 555 (Invitrogen, A32773) and Phalloidin-488 (1 : 100, Biolegend, 424 203) for F-actin staining, overnight at 4 °C. Lastly, after washing with 0.1% BSA in PBS thrice, nuclei were counterstained with DRAQ5™ (1 : 1000, Biolegend, 424 101) at RT for 15 min. Samples were kept in PBS at 4 °C in the dark until image acquisition. A Zeiss LSM880 confocal microscope was used to acquire *z*-stack images at 20× 0.8NA (0.42 μm per pixel) and 2 μm step size.

### 2.8. ORU volume quantification

After cell-laden microgel injection, collagen gelation and dextran degradation, osteoclasts were incubated for 2 h in a humified incubator at 37 °C and 5% CO_2_ and imaged at the Zeiss LSM880 confocal microscope. Reflection confocal microscopy with a 643 nm laser was used to obtain seven *z*-stack images of >50 μm per microfluidic chip at 20× 0.8NA (0.42 μm per pixel) and 2 μm step size. Each *z*-stack included at least one micro ORU. The coordinates of each image were saved so that each ORU could later be imaged again. Microfluidic chips were returned to the incubator and cultured for one week with α-MEM supplemented with 25 ng mL^−1^ rhM-CSF and rhRANKL, changing medium daily. After seven days, fixated samples were imaged with the same conditions at the coordinates used for imaging at day 0.

After image acquisition, a pixel classification workflow of the Ilastik toolkit^25^ (v. 1.4.0) was used for osteoclast and ORU segmentation with *σ* values of 0.7, 1.0, 1.6, and 3.5 for the Gaussian smoothing feature and 0.7, 1.0, and 1.6 for the remaining features: the Laplacian of Gaussian, Gaussian gradient magnitude, difference of Gaussian, structure tensor eigenvalues, and Hessian of Gaussian eigenvalues. As osteoclasts are also reflective, the Ilastik algorithm was trained to distinguish between cells and the collagen hydrogel. Up to 5 stacks with >25 images each were used in the pixel classification training. A custom ImageJ macro was used to obtain probability masks from the Ilastik segmentation workflow. Probability masks from osteoclast and ORU segmentation were converted to binary masks and merged together through image addition. The algorithm used to generate binary masks was selected by comparing the resulting masks with the ones generated by manual segmentation in six different slices from two independent image stacks (Fig. S1). The 3D Suite^26^ ImageJ plugin was then used to segment and quantify the volume of the ORU.

### 2.9. Statistical analysis

GraphPad Prism v10.4.2 was used for all statistical analysis. Unless otherwise stated in the figure legend, Mann–Whitney test was used to assess statistical significance, where differences between groups were considered significant when *p < 0.05, **p < 0.01, ***p < 0.001, ****p < 0.0001. All experiments were repeated as described in the figure legends.

## 3. Results

### 3.1. Structural features of bone remodeling units can be reproduced on-chip *via* microgels with controllable and predefined size

**Fig. 1:**
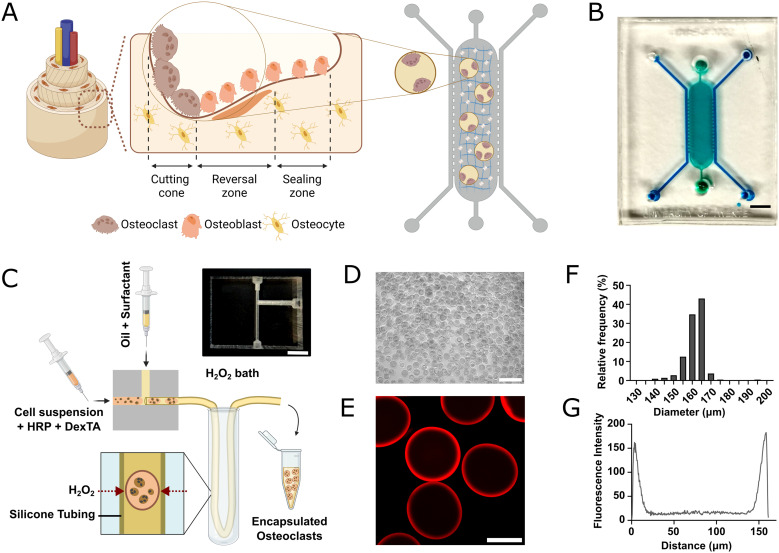
Structural features of BMU can be mimicked on-chip using hollow Dex–TA microgels. A) Schematic representation of bone remodeling units in cortical bone and matching ORU on-chip. Osteoclasts will be incorporated in ORU on-chip, within a mineralized collagen I matrix. B) Top view of the PDMS chip. A central chamber (7.5 mm × 2.4 mm × 0.25 mm) is flanked by two 0.5 mm wide perfusion channels, highlighted in green and blue, respectively. Scale bar – 2 mm. C) Schematic representation of the microfluidic droplet generator setup used to generate cell-laden hollow Dex–TA microgels *via* delayed outside-in crosslinking. Inset shows a photograph of the PMMA microfluidic device used to generate microgels. Scale bar – 1 cm. D) Micrograph of Dex–TA microgels after collection and washing. Scale bar – 400 μm. E) Confocal micrograph of Dex–TA microgel generated with 200 μm nozzles. Scale bar – 100 μm. F) Histogram of the distribution of Dex–TA microgel diameter (*n* > 200 microgels). G) Quantification of EtHD1 fluorescence across the microgel diameter. Spikes in fluorescence intensity indicate crosslinked Dex–TA, which can be used to quantify shell thickness.

In human cortical bone, bone remodeling takes place in specialized structures, the BMU, where mature osteoclasts degrade the bone matrix at the resorption front^1^ (Fig. 1A). The resorption front is semicircular with size in a range of 100–200 μm diameter.^2^ Aiming to reproduce structural features of native BMU, we envisioned an *in vitro* microfluidic system where mature osteoclasts would be introduced in mineralized collagen compartments with predefined size that matches the physiological dimensions of the BMU (Fig. 1A and B). To generate such compartments, we used a microfluidic droplet generator platform to generate monodisperse cell-laden microgels in high-throughput. In turn, these cell-laden microgels can undergo selective biorthogonal degradation, in which the hollow microgel is fully removed on-demand to release the cells into the chip. This strategy enables complete control over micro-architecture of the space the cells are confined to. In this study, Dex–TA polymer solution droplets were generated in flow focus mode, which were solidified *via* delayed enzymatic outside-in crosslinking using a previously described strategy^22^ (Fig. 1C). HRP-mediated Dex–TA crosslinking was induced by diffusion of H_2_O_2_ through a semipermeable tubing generating hollow microdroplets in high throughput (Fig. 1D and E). By flowing a 5% Dex–TA solution through a 200 μm nozzle at a total flow of 48 μL min^−1^ and water : oil ratio of 1 : 5, monodisperse hollow microgels with an average diameter of 161.8 ± 7 μm and average shell thickness of 8.95 ± 1.43 μm were generated (Fig. 1F and G).

**Fig. 2:**
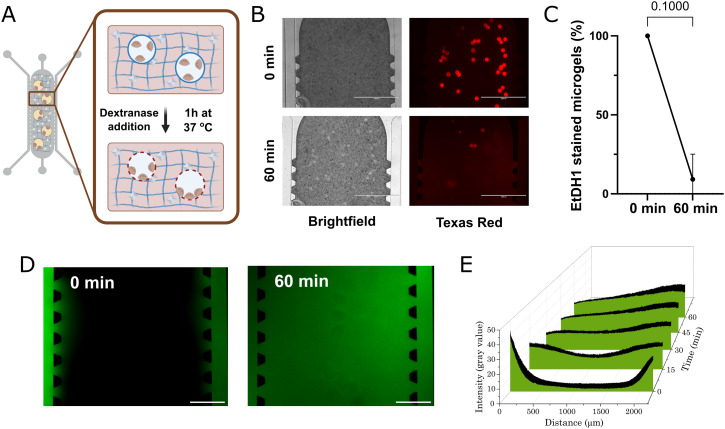
Generation of defined spherical hollow compartments through selective enzymatic degradation of Dex–TA microgels. A) Schematic depiction of dextranase driven ORU formation. B) Brightfield and fluorescence micrographs depicting microgel degradation after 60 min incubation with dextranase. Scale bar – 1 mm. C) Quantification of the number of intact Dex–TA microgels after 0 and 60 min dextranase incubation. Data from three independent experiments is expressed as mean ± SD (Mann–Whitney test, *p* = 0.1000). D) Fluorescent images depicting the diffusion of fluorescent molecules through HA-doped collagen hydrogels. After 60 min, FITC–dextran 10 kDa is able to diffuse through the entire hydrogel. Scale bar – 500 μm. E) Pixel intensity at longitudinal cross sections was plotted at different time points, over a total period of 60 min.

In our strategy, Dex–TA hollow microgels are used as vehicles for cell seeding, followed by biorthogonal degradation after collagen gelation, leaving compartments with the same volume as the original microgel (Fig. 2A). Thus, instead of embedding the osteoclasts in collagen matrix, this sacrificial step allows the generation of open compartments where osteoclasts can adhere to the surface of the matrix similar to what is observed *in vivo.*^2^ To demonstrate this approach, Dex–TA microgels were collected and seeded in HA-doped collagen hydrogels in our microfluidic device, and incubated with dextranase after collagen gelation. After 60 min incubation, dextranase diffused into the entire hydrogel compartment and selectively degraded the Dex–TA microgels, without affecting the surrounding collagen matrix (Fig. 2B). Approximately 90% of microgels were already degraded after 60 min of incubation, resulting in almost complete disappearance of EtHD1 signal (Fig. 2C). EtHD1 stains di-tyramine bonds and thus visualizes the crosslink density, its staining intensity correlates with Dex–TA microgel stiffness,^27^ which indicates that Dex–TA integrity is lost after dextranase treatment. To ensure that nutrients and metabolites are able to diffuse freely and support cell growth, we also confirmed that FITC–Dextran 10 kDa was able to quickly permeate the entire compartment within 15 min of incubation, even without active perfusion (Fig. 2D and E).

### 3.2. Human osteoclast differentiation status is maintained when encapsulated in ORU

**Fig. 3:**
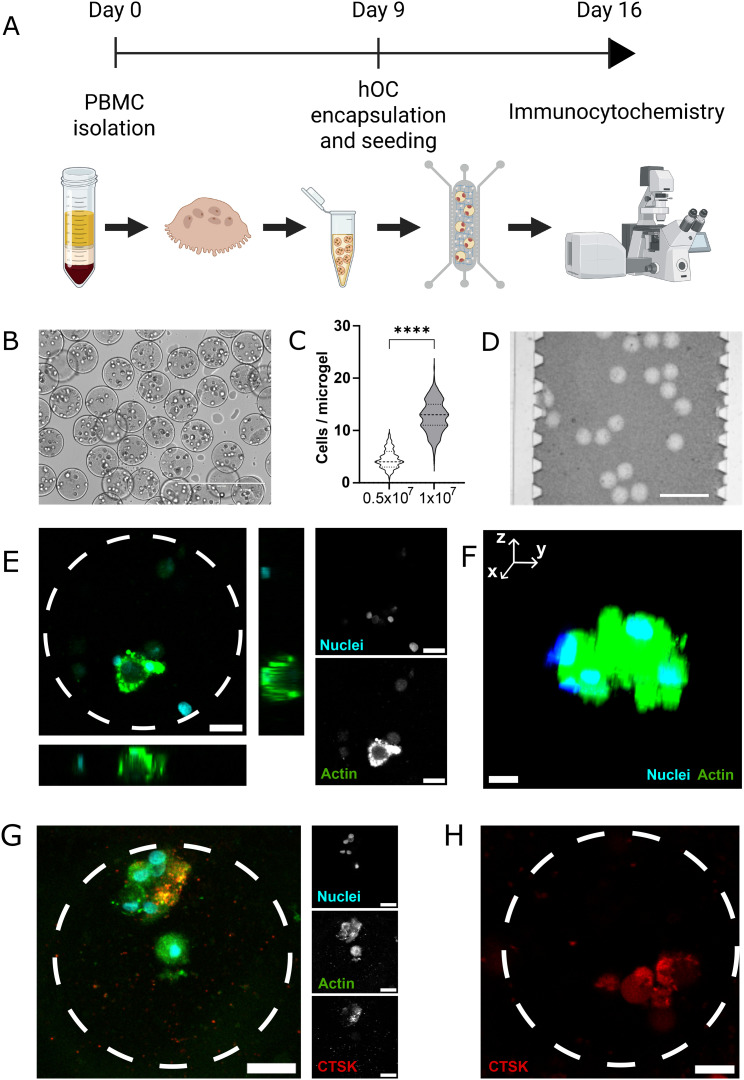
Osteoclast encapsulation, seeding and characterization on-chip. A) Experimental timeline. Osteoclast precursors were isolated from whole blood of healthy donors and differentiated for 9 days on cell culture flasks, followed by detachment and encapsulation in Dex–TA microgels and seeding on chips. Cells were cultured for one week and then fixated and stained for immunocytochemistry. B) Brightfield micrograph of cell-laden Dex–TA microgels. Scale bar – 400 μm. C) Quantification of cell concentration per microgel at two initial cell densities (cells per mL). Data from >370 microgels was expressed in violin plots (Mann–Whitney test, ****p < 0.0001). D) Brightfield micrograph of ORU on-chip (field of view representative from 3 independent experiments). Scale bar – 500 μm. E) Microphotograph of an osteoclast displaying characteristic features of a differentiated osteoclast including multinuclearity and an actin sealing zone (representative from 2 independent experiments). The middle point slice of a confocal stack and the respective orthogonal views (left panel) and individual color channels (nuclei – top, actin – bottom right). Scale bar – 20 μm. F) Three-dimensional rendering of the differentiated osteoclast. Nuclei – blue; actin – green. Scale bar – 5 μm. G) Maximum projection of a confocal micrograph of CTSK immunostaining (representative from 2 independent experiments). Dashed white circle denotes the border of ORU. Merged image (left) and individual channels (right). Nuclei – cyan, top; actin – green, middle; CTSK – red, bottom. Scale bar – 20 μm. H) Confocal micrograph of CTSK probe (representative from 2 independent experiments). Dashed white circle denotes the border of ORU. CTSK – red. Scale bar – 20 μm.

Primary human CD14^+^ monocytes were isolated from whole blood of healthy donors and differentiated in cell culture flasks for nine days (Fig. 3A). This protocol generates mature osteoclasts that are able to degrade bone slices *in vitro.*^28^ The differentiation step before encapsulation is intended to maximize the yield of mature osteoclasts that can be encapsulated in Dex–TA microgels, as the differentiated cell population is highly heterogeneous by nature. Osteoclasts were then detached and encapsulated in Dex–TA microgels, leading to the production of monodisperse microgels with an average of 12.6 ± 3.1 osteoclasts per microgel (Fig. 3B and C), consistent with the number of osteoclasts located in BMU *in vivo.*^29^ Varying the initial cell density during encapsulation changes the encapsulated cell number considerably (Fig. 3C). Microgels were then incorporated in HA-doped collagen, seeded in the microfluidic device, and cultured for seven days. Each chip contained on average 62 ± 20 ORU (Fig. 3D). A seven day time point was chosen to ensure cell attachment, differentiation and activity, which is in line with previous reports of osteoclast differentiation and maximum activity period.^30^ Using Dex–TA microgels as a vehicle for simultaneous cell encapsulation and seeding realizes both ORU generation and effective cell segregation in each ORU. After seven days of culture, characteristic features of differentiated osteoclasts were observed, including i) multinuclearity, ii) large cytoplasmic area, iii) actin ring and sealing zone formation, and iv) CTSK expression (Fig. 3E–G). Orthogonal views of the confocal stack, as well as 3D rendering of the osteoclast, show a clear sealing zone formation and defined structural organization. In addition, CTSK expression was not only confirmed by immunocytochemistry but also using specific CTSK fluorescent probes that generate red fluorescence after proteolytic cleavage by CTSK (Fig. 3G and H). Specifically, 92% of the ORU were characterized by CTSK expression/activity.

### 3.3. Osteoclasts degrade their surrounding matrix in the ORU

**Fig. 4:**
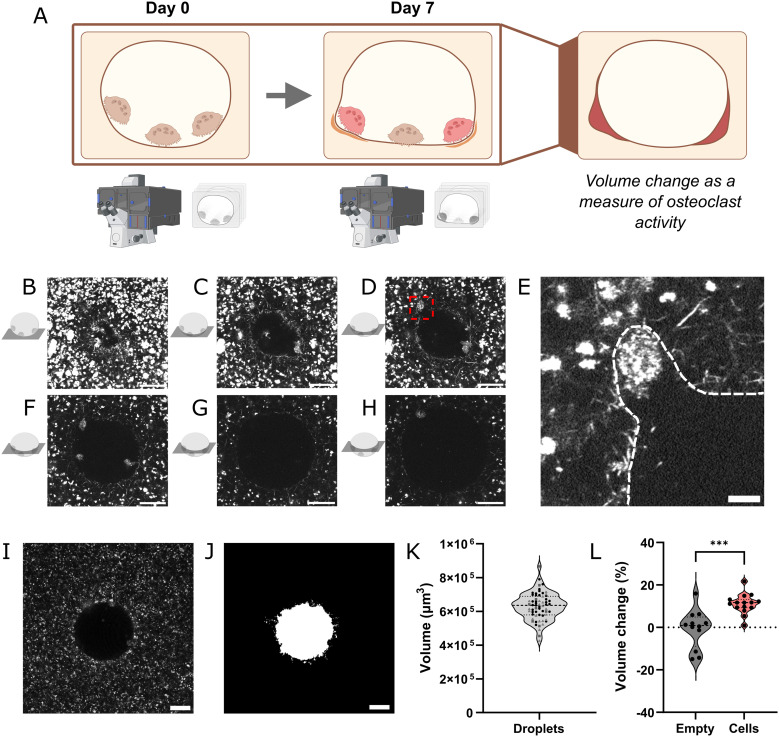
Quantification of ORU volume as a measure of osteoclast activity. A) Rationale behind the experimental setup and timeline. By quantifying the volume of ORU in the beginning and end of an experiment, the change in remodeling unit volume can be extracted and used as a measure of osteoclast activity. B) Micrographs of a representative ORU captured through reflection confocal microscopy imaged at 6 μm, C) 10 μm, D) 12 μm, F) 18 μm, G) 24 μm and H) 30 μm from the bottom of the ORU. Scale bar – 50 μm. E) Inset highlighting a zone of active matrix degradation of the image in D). The border of the ORU is shown in white dashed lines. Scale bar – 20 μm. I) Representative micrograph of a ORU and J) its respective segmentation for volume quantification. Scale bar – 50 μm. K) Volume distribution of ORU at day 0 of the experiment. Data from 63 ORU from three independent experiments is expressed as individual data points in violin plots. L) Quantification of volume change in empty or cell laden ORU. Individual data points from one independent experiment are expressed in violin plots (Mann–Whitney test, ***p < 0.001).

Increased bone resorption is a critical hallmark of osteoporosis. When modeling this disease *in vitro*, generating ORU with a predefined size presents an obvious advantage when quantifying the extent of osteoclast-driven matrix degradation. By monitoring the ORU volume at the initial and final time point of the experiment, the ORU volume change can be used as a measure of osteoclast matrix degradation (Fig. 4A). Nonetheless, volume quantification needs to be performed longitudinally, and hence a non-invasive quantitative method needs to be employed when imaging the ORU while limiting the hazardous effect of light sources on cell survival. As collagen matrices are highly reflective, confocal reflection microscopy enables label-free, 3D imaging of the entire ORU in under 2 min using a low energy 643 nm laser as light source. Micrographs of confocal stacks of a representative ORU are shown in Fig. 4B–H. As native collagen contains RGD motifs that facilitate cell attachment, osteoclasts are mainly found attached to the collagen surface after seven days of culture. Close inspection of the borders of the ORU reveals areas of apparent localized osteoclast activity, where cells are visible in pockets that are contrasting with the original smooth spherical shape of the ORU (Fig. 4E).

A region of interest was defined by analyzing each ORU within 50 μm from the glass coverslip. This decision stemmed from the fact that images of the bottom of the ORU, taken closer to the objective, are brighter and show increased contrast between the ORU and the surrounding HA-doped collagen matrix. As the distance from the bottom of the ORU increases, signal intensity decreases and distinguishing ORU and the matrix becomes challenging. In addition, most cells are attached to the bottom of the ORU due to gravity, and hence all events should be captured within this region of interest. We employed a machine learning algorithm to obtain an automatic and high-fidelity volume segmentation (Fig. 4I and J, Fig. S1). At the initial timepoint of the experiments, the average volume of the bottom 50 μm of the ORU was 6.29 ± 0.8 × 10^5^ μm^3^, which is consistent with the theoretical volume of a sphere with a diameter of 150 μm. As a proof-of-concept, we compared the volume differences between empty ORU and cell laden ORU and observed a significant increase in ORU volume when osteoclasts were present (Fig. 4L). As expected, the average change in volume of an empty ORU is close to zero, indicating that in static conditions the ORU remains stable throughout the duration of the experiment.

### 3.4. Increased activity of osteoclasts exposed to RANKL is recapitulated in ORU

**Fig. 5:**
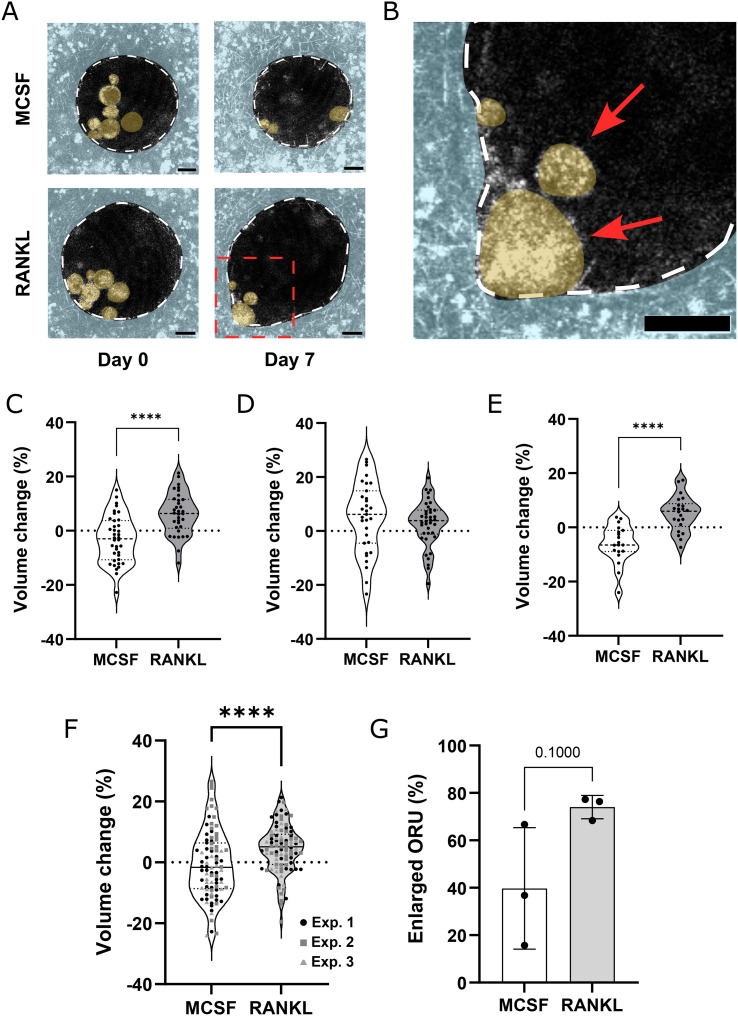
Increased ORU volume reflects the exacerbation of osteoclast activity following RANKL treatment. A) Representative micrographs of ORU from MCSF or RANKL treated samples. Images show the same ORU at the beginning and end of the experiment. The borders of the ORU are highlighted in white dashed lines. Cells and collagen hydrogels were artificially colored in yellow and light blue, respectively. Scale bar 20 μm. B) Inset of a ORU from RANKL treated samples at day 7. The border of the ORU is shown in white dashed lines and cells are highlighted in red arrows. Cells and collagen hydrogels were artificially colored in yellow and light blue, respectively. Scale bar – 20 μm. C) Quantification of volume change in MCSF and RANKL treated ORU in independent experiment one, D) two and E) three. Individual data points are expressed in violin plots (Mann–Whitney test, ****p < 0.0001). F) Quantification of volume change in MCSF and RANKL treated ORU. Individual data points from three independent experiments are expressed in violin plots (Mann–Whitney test, ****p < 0.0001). G) Percentage of ORU with increased volume after seven days of culture. Individual data points from three independent experiments are expressed as mean ± SD (Mann–Whitney test).

RANKL is a primary activator of osteoclast resorption *in vitro* and *in vivo.*^31,32^ To assess whether osteoclasts are responsive to external biochemical cues, we tested the effect of RANKL on osteoclasts cultured on ORU for one week. ORU were imaged by confocal reflection microscopy at day 0 and day 7 of the experiment (Fig. 5A). While a limited amount of matrix remodeling events were detected in control ORU, osteoclasts exposed to RANKL actively degraded the HA-doped collagen walls of ORU (Fig. 5B). After quantifying the ORU volume change from day 0 to day 7, we observed that RANKL stimulation led to a significant increase in volume of ORU, while control samples averaged close to zero (Fig. 5C–F). Interestingly, we observed inter-donor variability where osteoclasts sourced from one of the donors was less responsive to RANKL stimulation (Fig. 5C–E). Although small decreases in compartment volume during the course of the experiment can lead to negative volume change values, RANKL treatment consistently led to increased ORU volume (Fig. 5G).

## 4. Discussion

Despite the crucial importance of osteoclast resorption activity in the onset and development of osteoporosis and other skeletal disorders, it is often overlooked in currently available bone-on-chip OoC models developed to mimic these diseases. Among the limited number of bone-on-chip models incorporating osteoclasts, most primarily investigate their differentiation and interactions with other bone-resident cells, while providing limited insight into their matrix degradation activity.^18,19^ On the other hand, quantification of bone resorption in a bone-on-chip model still relies on labor intensive and time-consuming endpoint measurements.^33^ In the present work, we address these shortcomings by generating osteoclast-laden ORU with pre-defined highly reproducible micro-architecture. This feat enabled straightforward quantification of matrix degradation activity and it was amenable for non-invasive longitudinal monitoring for periods up to one week.

The cutting cone of the BMU in cortical bone is a site of active osteoclast-driven bone degradation. In humans, the tip of the cone is semicircular with size in a range of 100–200 μm diameter and populated by mature osteoclasts.^2^ While cellular components are crucial for disease modeling, replicating this structural arrangement was the base motivation for our strategy for bone tissue biomimicry on-chip. As such, to generate precisely defined micro-compartments, we used enzymatically degradable, monodisperse cell-laden microgels seeded in a mineralized collagen matrix. Fabricating microgels with a pre-defined size of ∼150 μm allowed for striking a balance between the initial cell density required and the resulting cell numbers in each microgel, to a level that is similar to that *in vivo.*^2^ Microfluidic droplet production is an established technology, capable of generating monodisperse microgels with controllable size and thickness.^8,21–23,27^ Droplet generators were previously used to encapsulate osteoclast precursors for high-throughput osteoclast differentiation and delivery.^22^ Herein, a different strategy was employed to instead deliver mature osteoclasts *via* sacrificial hollow Dex–TA microgels together with a HA-doped collagenous matrix into a microfluidic chip. We then took advantage of selective dextran degradation to release the osteoclasts into ORU with a defined size. Similar microparticle sacrificial strategies have been employed to generate alveolar lung-on-chip, where packed alginate microbeads are infiltrated with gelatin methacryloyl and then removed by incubation with ethylene diamine tetraacetic acid after hydrogel crosslinking.^9^ This generates interconnected micropores that are then fully covered with cells. Nonetheless, to mimic BMU, ORU coupling is not necessary and Dex–TA microgels allow for simultaneous cell–hydrogel seeding, while keeping a tight control over ORU cell density. Microfluidic droplet generators have also been used to establish co-culture systems with different cell types,^34^ which in the future could be applied to expand the complexity of our model by including osteoblast–osteoclast co-cultures in each ORU.

The ability for continuous monitoring of culture parameters and cellular behavior while replicating key pathophysiological features is one of the main advantages of using OoC models.^35,36^ Nevertheless, the limited sample volume characteristic of these systems often does not allow sufficient sample recovery for reliable quantification using conventional molecular biology assays. Image-based assays are commonly used for characterization and measurement of cellular differentiation and activity. However, when modeling bone, the characteristics of mineralized bone ECM hamper the use of traditional imaging techniques for routine inspection, particularly its opacity. Micro-computed tomography is commonly used to track bone remodeling events *in vivo*,^37,38^ but its application to bone-on-chip models has not yet been demonstrated. Other strategies to effectively track bone cell activity include the use of two-photon microscopy that presents higher tissue penetration.^39^ Nonetheless, such techniques require pre-labelling of cells, which is challenging when using human primary cells susceptible to phototoxicity. Here, we used label-free, low-energy confocal reflection microscopy to measure the volume of ORU and estimate osteoclast activity longitudinally. We took advantage of both the high reflectivity of collagen matrices as well as the predefined size and shape of Dex–TA microgels to identify and measure the change in volume of ORU. Matrix remodeling events are identifiable by comparing the volume at the beginning and end of the experiment. In addition, using Dex–TA microgels as a vehicle for osteoclast transfer and ORU generation allows for increased throughput, with more than 60 ORU per chip. In the future, one could envision coupling of our chip to a live-imaging system with environmental control to further increase the number of ORU analyzed in each independent experiment.

Another crucial component to our analysis workflow is the automatic segmentation of ORU volume. Imaging ORU generates a high volume of data that becomes cumbersome and time-consuming to manually segment. To circumvent this issue, we applied an Ilastik toolkit to automatically segment ORU volume with high accuracy. Previously, other machine learning algorithms have been used to assess osteoclast differentiation *in vitro*,^28,40,41^ but to our knowledge this is the first time such tools have been used to assess osteoclast activity in bone-on-chip platforms. Ilastik is also easily integrated with custom ImageJ macro language to couple the pixel classification algorithms to commonly used thresholding and segmentation algorithms, allowing for generation of segmented ORU in 3–5 minutes. This represents a 70% reduction in processing time while also reducing bias inherent to manual segmentation. Nevertheless, it should be noted that image acquisition parameters such as image size, exposure time, and gain can affect the quality of segmentation. Brightness, edge, texture, and other pixel features affect classifier categorization in Ilastik.^25^ Therefore, translation of our segmentation model to other workflows will require re-training and validation unless the same equipment and image acquisition parameters are used to image the ORU.

Segmentation of ORU volume allowed us to decipher the effect of RANKL activation of osteoclast activity, as RANKL-treated ORU displayed increased volume change after seven days of culture. Treatment with RANKL resulted in an average increase in ORU volume of approximately 7%. *In vitro*, osteoclast resorption activity is often quantified by seeding osteoclasts on top of bone slices,^42^ calcium phosphate coated well plates,^43^ or commercial assays such as osteo assay surface.^44^ Quantification of osteoclast activity in these assays is image-based, where RANKL treatment has shown to induce an increase in resorbed area from ∼2% to 35% depending on the assay used as well as the osteoclast seeding and differentiation conditions. Bone slices offer the clear advantage of preserving the physicochemical cues of native bone; however, resorption quantification is an endpoint assay, and its outcomes are inherently influenced by the operator's experience.^40^ On the other hand, calcium phosphate coated well plates provide an easy and standardized assay, but they lack organic components of the bone matrix and are limited to the assessment of osteoclast demineralization activity. Although combining droplet microfluidics with OoC technology is complex, ORU have clear advantages compared to the other resorption assays. ORU are embedded in a collagen I matrix doped with HA and vitronectin, which together provide the RGD motifs that support integrin-mediated adhesion and signaling, as well as a mineral phase essential for osteoclast polarization and activity.^45,46^ In addition, ORU provide a non-invasive and semi-automatic tool for screening changes in compartment volume as a measure of osteoclast activity, allowing for the tracking of volumes throughout the course of the experiment, while reducing sample processing time and measurement bias.

This is the first demonstration of the applicability of ORU as a model system to study osteoclast activity in bone-on-chip models. Importantly, we found variability between donors of osteoclast precursors, where osteoclasts in one of the independent experiments were less responsive to RANKL. As osteoclast encapsulation and microgel loading is reproducible across independent experiments, we expect that inherent differences in osteoclast donors drive a considerable fraction of the variability observed. This variability is both expected and desirable, as the activity of primary human osteoclasts is highly variable depending on donor age, sex, lifestyle habits, for instance.^47–49^ Assessing the effect of donor demographics was out of the scope of the current work, but it is an integral component when considering the use of ORU for inclusion in drug discovery pipelines. We also observed that several ORU, both cell-free and cell-laden, displayed negative volume change percentages in our experiments. Collagen hydrogels are prone to contraction when in contact with cells, but even in cell-free hydrogels slight variations in size are observed after one week.^50,51^ Moreover, shear forces imposed by fluidic flow, (*e.g.* during medium change), can also contribute to changes in hydrogel shape.^52^ In the future, we intend to explore different hydrogel compositions and strategies to improve hydrogel mechanical stability and increase the experimental timeframe during which ORU are stable. This will allow the inclusion of osteoblast-lineage cells, co-delivered with osteoclasts inside ORU or embedded within the collagen matrix.

The study represents a first step towards establishing a BMU model, with osteoclasts as a focus point. Nevertheless, osteoclast activity is known to be modulated by a multitude of other cellular players that are located within the BMU milieu.^53^ Osteoblast-lineage cells play a critical role in the coordination of osteoclastogenesis and bone resorption, through direct cell–cell stimulation as well as *via* secretion of paracrine factors including RANKL.^54–57^ Additionally, vasculature development occurs concurrently with osteoclast-mediated bone resorption, leading to continuous crosstalk between osteoclasts and endothelial cells.^58^ Not only are blood vessels important for the recruitment of osteoclast precursors to the BMU, but also the secreted factors and resorption products generated by osteoclasts act as angiogenic factors that drive neovascularization.^2^ Thus, the addition of osteoblast-lineage cells and endothelial cells would further increase the biomimicry potential of our model. Previous studies have developed bone-on-chip devices with microvascular networks in HA-doped fibrin hydrogels,^59^ and similar strategies could be applied in the next iterations of the model to generate vascularized ORU models with increased translational power. In the future, the establishment of vascularized, osteoblast/osteoclast laden ORU-on-chip would be a key steppingstone towards reaching the complexity required for modelling bone.

## Conclusions

We herein combined two microfluidic technologies, a droplet generator and an OoC model, to mimic events taking place during bone remodeling. We leveraged enzymatically degradable Dex–TA microgels to develop a BMU inspired bone-on-chip system featuring confined, osteoclast laden microstructures directly embedded in mineralized collagen. The pre-defined micro-architecture of human osteoclast-laden Dex–TA microgels confers an advantage for mimicking the physiological features of BMU, as well as establishing the baseline volume for matrix degradation quantification. Human osteoclasts were morphologically and functionally differentiated on-chip, and their activity was estimated through the non-invasive and quantitative analysis of individual ORU. Moreover, the throughput was enhanced as a result of the integration of multiple, individually addressable ORU in each chip. The ORU-on-chip model described here addresses critical limitations of traditional bone-on-chip models and was applied in a proof-of-concept experiment to demonstrate increased matrix degradation in RANKL treated osteoclasts on-chip.

In summary, our strategy combines precise and reproducible droplet generation with the biological relevance of bone-on-chip platforms, and presents an opportunity for development of bone remodeling-on-chip with a focus on human osteoclast activity. We anticipate that the highly defined micro-niches of ORU-on-chip models will be valuable for both fundamental studies of bone disease mechanisms and integration into drug discovery pipelines.

## Author contributions

Francisco Conceição: conceptualization, methodology, validation, formal analysis, investigation, writing – original draft, review & editing, visualization. Nuno Araújo-Gomes: methodology, investigation, writing – review & editing. Johanna F. A. Husch: methodology, investigation, writing – review & editing. Malin Becker: methodology, writing – review & editing. Jeroen J. J. P. van den Beucken: resources, supervision, writing – review & editing. Jeroen Leijten: supervision, writing – review & editing. Liliana Moreira Teixeira: writing – original draft, review & editing, supervision, project administration, funding acquisition.

## Conflicts of interest

The authors declare that they have no known competing financial interests or personal relationships that could have appeared to influence the work reported in this paper.

## Supplementary Material

LC-026-D5LC00682A-s001

LC-026-D5LC00682A-s002

LC-026-D5LC00682A-s003

## Data Availability

All raw and processed data presented in this manuscript has been uploaded and stored in institutional data servers where it is securely maintained under the direction of the corresponding author. All data, raw and processed, can and will be made available to any interested parties. They are encouraged to directly contact the corresponding author (Dr. Liliana Moreira Teixeira, l.s.moreirateixeira@utwente.nl) to request access to the data. Supplementary information (SI) is provided to illustrate the automatic segmentation workflow used for ORU volume quantification. SI is available at the following DOI: https://doi.org/10.1039/d5lc00682a.
