# Supplementary figures and images for: Engineering osteoclast resorption units via sacrificial microgels in a bone-on-chip platform
Source: Lab Chip. 2025 Nov 11;26(2):331–44. doi: 10.1039/d5lc00682a (PMC12624845; doi:10.1039/d5lc00682a)

A

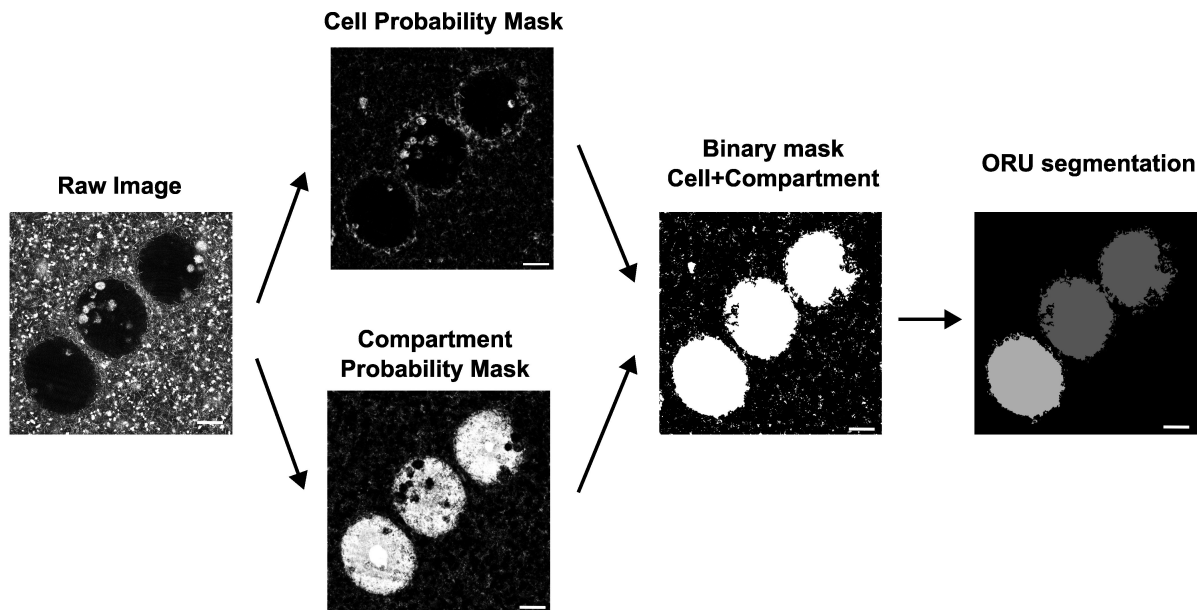

B

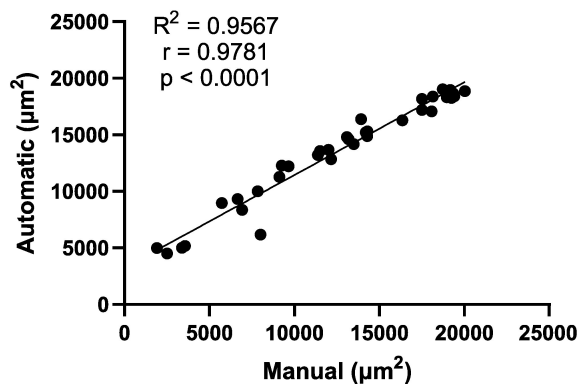

Supplement: LC-026-D5LC00682A-s002 [file LC-026-D5LC00682A-s002.pdf]
