# Supplementary material for: Engineering osteoclast resorption units via sacrificial microgels in a bone-on-chip platform
Source: Lab Chip. 2025 Nov 11;26(2):331–44. doi: 10.1039/d5lc00682a (PMC12624845; doi:10.1039/d5lc00682a)
Supplement: LC-026-D5LC00682A-s003 [file LC-026-D5LC00682A-s003.pdf]

Supplementary Information (SI) for Lab on a Chip.
This journal is © The Royal Society of Chemistry 2025

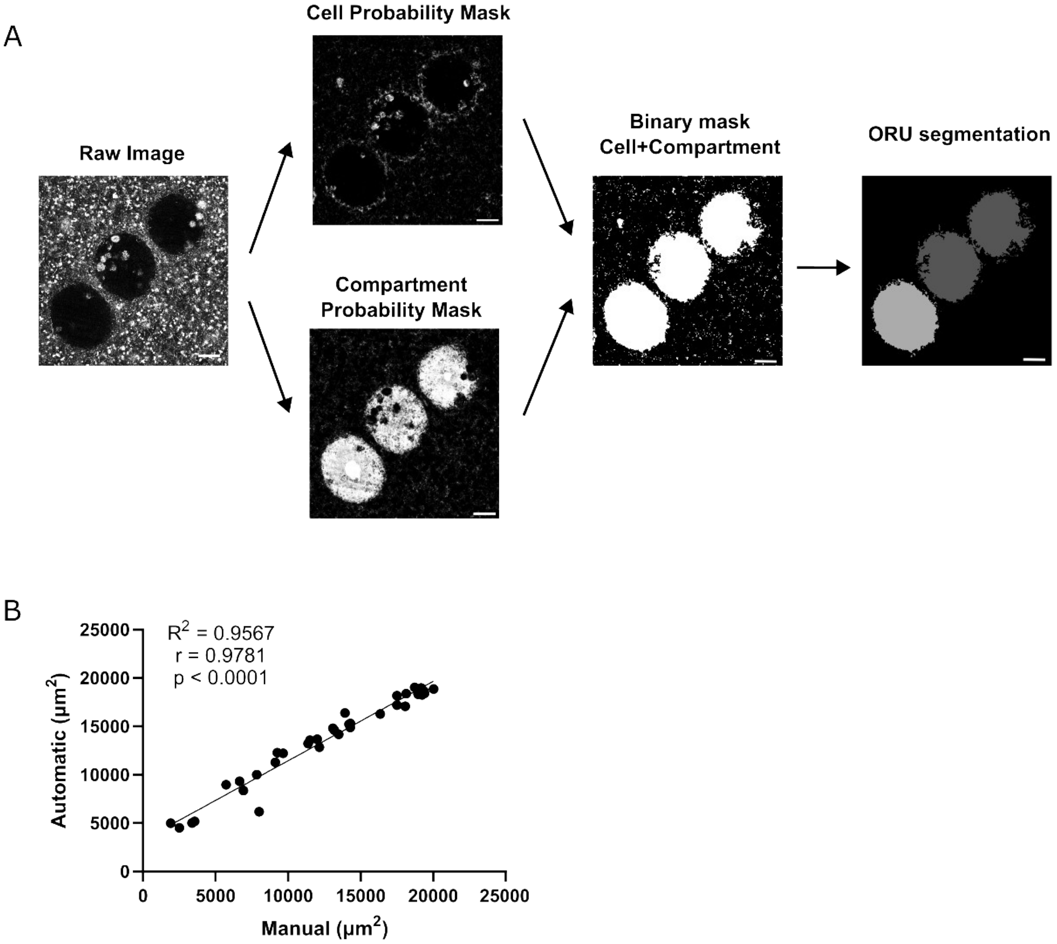

Figure S1. Automatic segmentation of ORU using an Ilastik pixel classification algorithm. A) Workflow of ORU segmentation. The pixels in raw images are classified using machine learning and probability masks are generated for the ORU compartment and osteoclasts. In FIJI, binary masks are obtained for both components and added to obtain the binary mask for the whole ORU, followed by segmentation and volume measurement. Scale bar - 50µm. B) Linear regression of ORU volume obtained through manual and automatic segmentation.
